# The Physiology of Obese-Hyperglycemic Mice [*ob/ob* Mice]

**DOI:** 10.1100/tsw.2007.117

**Published:** 2007-05-29

**Authors:** Per Lindström

**Affiliations:** Department of Integrative Medical Biology, Section for Histology and Cell Biology, Umeå University, S-901 87 Umeå, Sweden

**Keywords:** obese hyperglycemic, mice, leptin, pancreatic islets, insulin resistance, immunity, reproduction

## Abstract

This review summarizes key aspects of what has been learned about the physiology of leptin deficiency as it can be observed in obese-hyperglycemic *ob/ob* mice. These mice lack functional leptin. They are grossly overweight and hyperphagic, particularly at young ages, and develop severe insulin resistance. They have been used as a model for obesity and as a rich source of pancreatic islets with high insulin release capacity. The leptin deficiency manifests also with regard to immune function, the cardiovascular system including angiogenesis, supportive tissue function, malignancies, and reproductive function. *ob/ob* Mice are well suited for studies on the interaction between leptin and insulin, and for studies on initial aspects of metabolic disturbances leading to type-2diabetes.

## INTRODUCTION

The mouse *ob/ob* syndrome was discovered in 1949 in an outbred colony at Roscoe B. Jackson Memorial Laboratory, Bar Harbor, Maine[1] and was transferred to the already well-characterized C57Bl mice colony that had been established during the 1930s. *Ob/ob* mice (OM) are hyperphagic, obese, hyperinsulinemic, and hyperglycemic[2,3], and they were used as a model for diabetes and obesity. The pancreatic islets are large and contain a high proportion of insulin-producing ß-cells. OM have, therefore, been used as a source of pancreatic islets for studies of ß-cell function. It was soon discovered that OM have a number of other traits in addition to obesity. They are, for example, infertile and have impaired immune functions.

Elegant parabiosis experiments showed that OM lack, but are very sensitive to, a circulating factor produced by their normal siblings[4,5]. By extensive positional cloning experiments, this factor was identified in 1994 by Friedman and coworkers as leptin produced in adipose tissue[6,7]. The *ob/ob* syndrome can be reversed almost completely even in adult animals by exogenous leptin or transfection with the leptin gene[8,9,10]. There are also cases with leptin deficiency in obese humans, but this is uncommon, so OM do not present a good model for the etiology of human obesity[11]. However, the discovery of leptin has opened up a whole new field of studies on regulation of food intake, metabolic turnover, and obesity. We also have learned a lot more about the interrelationship between metabolism and other functions, such as reproduction and the immune system.

Insight into the physiology of leptin has been achieved through studies in humans and several animal models, including rodents with leptin receptor defects, such as *db/db* mice and *fa/fa* rats[12,13]. This review will focus on observations in OM. In particular, OM with a relatively mild syndrome will be discussed (see under Metabolism below). The cellular mechanisms for the effects of leptin have been the subject of excellent reviews elsewhere[14,15]. The complex neuropeptidergic control of food intake and metabolic rate in the hypothalamus and other parts of the central nervous system (CNS) is under intense investigation, as well as the connection between metabolic and other hormone-regulated functions. There are also many recent reviews on this[16,17,18,19]. Leptin signaling and insulin-leptin-neuropeptidergic interactions will be referred to, but not covered in any detail, in this presentation.

OM are indistinguishable from their lean littermates at birth, but within 2 weeks, they become heavier and develop hyperinsulinemia. These differences are much more pronounced after weaning and overt hyperglycemia is observed during the fourth week. The blood glucose rises to reach a peak after 3–5 months when the mice also have a very high food intake and a rapid growth[20,21,22]. After that, blood glucose values decrease and eventually become nearly normal at old age. Insulin levels peak later[20]. The animals remain insulin resistant, but impaired glucose tolerance and glycosuria after a glucose load is observed mostly in the postweaning period of rapid growth, and this usually becomes normalized when the mice get older[20,23,24,25].

## PANCREATIC ISLETS

The islet volume in OM is up to ten times higher than in normal mice[26,27] and insulin-producing ß-cells are by far the most numerous[20,27,28,29]. The islet hyperplasia is probably not caused by a primary abnormality in the islets due to leptin deficiency; it is rather the consequence of an increased demand for insulin. The growth may be triggered by hyperglycemia, but also by other bloodborne factors and is evident from the fourth week[22]. Persistent hyperglycemia indicates that ß-cell function is insufficient despite hyperinsulinemia, but ß-cells from many OM strains have a high capacity to secrete insulin. After an overnight fast, the blood glucose is nearly normalized and OM islets release larger quantities of insulin after fasting when compared with normal mouse islets[30]. Islets from OM also respond adequately to stimulators and inhibitors of insulin release in most experimental conditions[31,32]. They have therefore been used in several hundred papers as a rich source of ß-cells in studies of islet function.

ß-Cells have leptin receptors and leptin inhibits insulin release in most studies[33,34]. This may explain some of the functional differences between ß-cells from OM and normal mice. The ß-cells also become insulin resistant[35,36] and the difference from lean mouse ß-cells can be viewed as direct adaptations to the OM syndrome with leptin deficiency combined with hyperinsulinemia and hyperglycemia. The threshold for glucose-induced insulin release occurs at a lower glucose concentration[30,37]. The mechanisms for this may, in part, be similar to the glucose hypersensitivity observed after prolonged exposure to elevated glucose also in islets from normoglycemic animals, and involve both metabolic and ionic events[38,39]. Insulin resistance is coupled to a reduced phosphatidylinositol 3-kinase (PI3K)–dependent signaling pathway and a reduced PI3K may result in disinhibition of glucose-induced insulin release[36]. Glucose-6-phosphatase activity is higher in islets from OM[40]. ß-Cells from OM are more sensitive to the stimulatory effect of acetylcholine and the inhibitory effect of noradrenalin[41], but there is age dependence for this. Islets from young OM have an increased ß-cell responsiveness to cholinergic stimulation already from 10–12 days of age[42], but the sensitivity to acetylcholine is reduced at old age, whereas the sensitivity to vagal neuropeptides may be increased[43,44]. Islets from OM respond with a larger increase in insulin release within 10 min after stimulation with ACTH 1-39[45]. OM ß-cells have increased activity of uncoupling protein-2 that may reduce insulin release[46], but knockdown of UCP-2 expression had no effect on glucose-induced insulin release in OM islets[46]. OM islets have a reduced capacity to accumulate cAMP[47,48], but they are more sensitive to a rise in cAMP[48]. One effect of cAMP could be to reduce the inhibitory effect of a rise in UCP-2[49]. OM ß-cells have an increased Na/K-ATPase activity[50] and may be more sensitive to voltage-dependent events[51], but an impaired function of voltage-dependent $Ca^{2+}$ channels[52] and a disturbed pattern of cytoplasmic calcium changes after glucose stimulation has also been reported[53]. They also do not show the same type of cell-specific $Ca^{2+}$ responses that are found in lean mouse islets[54]. There is an excessive firing of cytoplasmic $Ca^{2+}$ transients when OM ß-cells are stimulated with glucagon[55]. Islet amyloid polypeptide is increased in islets from OM[56]. OM islets also have a reduced capacity to increase blood flow to meet metabolic demands[57].

Glucagon levels are high in OM[58]. It was early hypothesized that elevated glucagon secretion contributes to the altered metabolism of OM[59] and immunoneutralization of endogenous glucagon improves metabolic control[60]. There is a correlation between serum glucagon levels and hepatic glucose output in type-2 diabetic patients[61] and reduction of serum glucagon may be a target for diabetes treatment.

One of the features of OM is that they have large pancreatic islets consisting of mostly ß-cells and OM islets have been used in studies of ß-cell proliferation. There is a good correlation between the level of hyperglycemia and islet cell replication in rats[62] and obese-hyperglycemic mice[63], and the morphology of OM islets reaggregated *in vitro* depends on the glucose concentration[64]. ß-Cell growth is probably stimulated by hyperglycemia directly or indirectly. It has been suggested that cells recruited from bone marrow increase the insulin release capacity in OM[65]. It is also possible that duct progenitor cells are involved in the expansion of the ß-cell mass, but mitotic figures have been demonstrated in ß-cells from OM[22,27] and cells within existing islets are presumably the most important sources for islet cell hyperplasia during expansion of the total islet mass[66]. OM have a growth-promoting environment for ß-cells depending on extra pancreatic factors[67,68] and oncogenes stimulate OM ß-cell replication as a sign that they can be manipulated extrinsically[69]. Bloodborne factors involved probably include NPY[70] and GLP-1[22,71], which both stimulate OM ß-cell replication.

## METABOLISM

The most obvious characteristic of leptin-deficient OM is that they are grossly overweight and have high food consumption. They are also hyperglycemic, hyperlipidemic, and hyperinsulinemic. They have a disturbed thermoregulation and lowered physical activity. The lower energy expenditure gives OM an increased food efficiency to accumulate fat, and they have been used in many studies of the effects of antiobesity and antidiabetogenic drugs. Both increased food intake and reduced energy expenditure are direct consequences of leptin deficiency. OM are by definition an excellent model of leptin deficiency. They are perfectly suited for studies of the interaction between leptin and insulin on metabolic functions (in mice), but OM have also been used extensively as a more general model for obesity, insulin resistance, and lipotoxicity. The list includes diabetes. OM may be a model for prediabetes, but the insulin release capacity remains high throughout life[72,73]; the hyperglycemia is reduced after 6 months of age[20]. OM develop peripheral neuropathy[74], but the severity of diabetes complications, such as kidney damage, is much lower than in *db/db* mice[75]. The OM is therefore probably not a good model for all aspects of manifest type-2 diabetes. It has also been questioned whether the effect of leptin to increase energy expenditure applies to humans[76].

It was soon recognized that the *ob/ob* syndrome varies considerably depending on the genetic background[77,78]. In this presentation, “OM” refers to 6J or Umeå *ob/ob* mice unless otherwise stated. On a 6J background, hyperglycemia is relatively mild, particularly at old age, and glycosuria is usually not present in the fasting state. On a KsJ or BTBR background, the mice become overtly diabetic with a reduced life expectancy[4,79]. These mice have a higher food intake than OM on a 6J background[80]. One difference between 6J and KsJ OM is that 6J mice have a larger lipogenic capacity in the liver[81], which may render them less susceptible to lipotoxic effects. ß-Cells from OM accumulate fat, but only a small lipid increase is observed in ß-cells from OM on a 6J background[82], which is in keeping with the better-preserved function. There are also differences between individual mice from the same colony of 6J and Umeå *ob/ob* strains with regard to hyperglycemia and other aspects of a "diabetes-like" condition. This has been used also to select subgroups of animals within the same strain for metabolic studies.

Food intake is enhanced in OM because of leptin deficiency. Leptin suppresses orexigenic neuropeptide Y (NPY) in the hypothalamus and increases anorectic signals[12]. OM have been a model to study the effect of leptin on food intake, but many observations show clearly that other regulatory systems must also be involved. Insulin is a well-known regulator of food intake[17,18], although this has not been studied much in OM. Dopamine agonists[83,84] reduce food intake and almost reverse the metabolic aspects of the syndrome. Serotonin and serotonin uptake inhibitors reduce food intake and lower the hyperglycemia[85,86]. Other substances that affect food intake in OM in the absence of leptin include antiepileptic drugs[87], cannabinoid receptor agonists[88], peroxisome proliferator-activated receptor activators[89,90,91], endotoxin[92], intracerebroventricular noradrenalin and 5-HT[86], and thyroid hormone[93]. It is very likely that many of these agents exert their effects through the same neuropeptidergic systems as leptin.

OM have a lower body temperature than lean mice and a reduced thermogenic response to cold[94,95,96]. Leptin increases energy expenditure through both central and peripheral effects. Studies with leptin treatment of OM[97,98] and NPY knockouts in OM[99] strongly support that perhaps the most important central effect of leptin is to lower NPY release in the hypothalamus. NPY stimulates food intake, but also inhibits brown adipose tissue thermogenesis[100]. There are many signs of abnormal heat production in brown adipose tissue (BAT) and white adipose tissue (WAT) in OM[101,102]. Uncoupling protein-1 is decreased in both BAT and WAT[103]. PPARγ coactivator-1 (PGC-1) is important for cold-induced up-regulation of UCP-1. PGC-1 is low in OM, and leptin up-regulates PGC-1 in BAT and WAT both *in vivo* and *in vitro*[104]. OM have an impaired capacity to increase the level of thyroxin-dependent enzymes in response to cold[105]. Leptin also induces thermogenesis through increased lipid oxidation independently of uncoupling protein[106].

The main signaling pathways for leptin are the JAK/STAT transduction cascade, the mitogen-activated protein kinase (MAPK) cascade, the PI3K, and the 5'-AMP-activated protein kinase (AMPK) pathways[14,15]. There are different isoforms of the leptin receptor. The full-length leptin receptor is required for the JAK/STAT response, and is present in adipose tissue, muscle, liver, and pancreatic islets[14,15,16,17,18,19]. However, in skeletal muscle, the shorter receptor form, which activates PI3K, is predominant[107]. Leptin signaling pathways may interact with insulin signaling at several points including JAKs, PI3K, and MAPK[108]. This interaction between insulin and leptin is complex and tissue dependent[107,108,109,110], but studies in OM clearly indicate that the net effect of leptin is to increase insulin sensitivity[107,111] and that leptin resistance worsens insulin resistance. High caloric intake and absence of leptin can therefore be partly independent causes of insulin resistance in OM. Obese individuals are usually both insulin resistant and leptin resistant. However, the total absence of leptin signaling from the onset of obesity in OM is in sharp contrast to obesity in humans, and the cross-talk between the cellular effects of insulin and leptin is obviously absent.

Insulin resistance in skeletal muscle is probably central for the metabolic disturbances leading to type-2 diabetes and may be caused by intramyocellular lipid accumulation, which reduces muscle glucose uptake and mitochondrial function[111,112]. Most mechanisms thought to be of importance for the development of insulin resistance have also been observed in OM. OM have increased myocellular lipid content in heart[113,114] and skeletal muscle[115]. Insulin binding and IRS-1 activation is reduced[116,117], and OM skeletal muscle cells have an impaired glucose uptake and glycogen synthesis[116,118]. Glucose transporter (GLUT) 4 is the major glucose transporter of muscle and adipose cells and GLUT-4 is regulated by insulin through post-translational events. Adipocyte GLUT-4 is decreased in OM[119], but not the muscle GLUT-4 protein concentration[120]. GLUT-4 in the arcuate nucleus is higher in OM, particularly at the level of the plasma membrane, which indicates a high glucose uptake[121]. Gluconeogenesis is increased in OM[122,123,124] and is not related to fasting to the same extent as in lean mice[125]. The gluconeogenesis in OM also involves mechanisms that contribute very little in lean mice, e.g., gluconeogenesis from serine[126]. Muscle protein catabolism is enhanced in OM[127]. Mitochondrial genes involved in muscle mitochondrial respiration are up-regulated in diabetics, but only few of those enzymes are up-regulated in OM[128] and oxygen consumption is reduced in OM muscle fibers[129]. An example of the complexity of the interaction between leptin and insulin is that leptin has a direct inhibitory effect on glycogen synthesis in OM soleus muscle[130].

The muscle insulin resistance is not observed in very young mice, but develops after 3–4 weeks[131]. There is also an age dependence for hepatic and myocardial lipid content that parallels blood glucose and body weight, i.e., an increase in young mice followed by a decrease in old mice, although serum insulin levels are increased throughout life[132,133,134]. The rise in lipogenesis occurs earlier than insulin resistance in some studies[135]. The lack of leptin renders OM muscle tissue unresponsive to changes in body weight and exercise[136].

Mechanisms that can contribute to insulin resistance include the rise in inducible nitric oxide synthase in OM muscle and liver cells[137,138], and the increase in glucose flux via the hexosamine pathway in muscle[120]. Leptin has a direct effect *in vitro* on OM skeletal muscle cells to oppose the lipid incorporating effect of insulin. This improves, but does not correct, insulin resistance[139]. Reduced mitochondrial function may lead to intracellular fat accumulation and lipotoxicity. Mitochondria are impaired in OM adipose tissue[140], liver[141], and skeletal muscle[128,142], but also, for example, in macrophages[143]. Increased lipid peroxidation in the vicinity of mitochondria has been implicated in the pathogenesis of lipotoxicity and OM have increased production of hepatocyte reactive oxygen species[144]. Inhibition of lipid peroxidation reduces liver cell damage in OM[145]. Resistin levels may be high in OM and this can also worsen the insulin resistance[146].

Leptin signals through cytokine receptor–like signals including the JAK/STAT pathway and STAT3 is down-regulated in arcuate nucleus of OM[147]. Leptin also increases JAK/STAT signaling in OM in peripheral tissues[148]. The JAK/STAT pathway is under negative-feedback control by suppressors of cytokine signaling (SOCS). Hypothalamic and liver cell SOCS is induced by leptin in OM[149,150], but SOCS-3 mRNA levels are increased also in untreated OM[151]. Because OM are highly leptin sensitive, the latter observation would speaks against SOCSs being important for the development of leptin resistance, but most likely the increase in SOCS is related to insulin resistance.

Another common pathway for leptin and insulin regulation of metabolism is PI3K activation, which regulates nitric oxide synthase and cAMP formation. Both leptin and insulin stimulate PI3K. PI3K activity is reduced in OM[152] and insulin stimulation of PI3K in muscle is almost absent[153]. The low PI3K activity in OM ß-cells has been suggested to explain the reduced effect of insulin to inhibit insulin release[36].

Protein tyrosine phosphatase 1B (PTP1B) is a negative regulator of both leptin and insulin signaling and overexpression of PTP1B causes leptin resistance[154]. PTP1B levels are increased in OM liver cells[155] and OM has frequently been used for the characterization of PTP1B inhibitors[156]. Leptin up-regulates PTP1B expression in OM liver cells[157]. Probably the leptin-induced increases in both SOCS and PTP1B serve to maintain insulin and leptin signaling in balance.

The leptin receptor shows structural and functional homology with receptors for several cytokines that lack intrinsic kinase activity including IL-6. Serum IL-6 is increased in OM[158] and IL-6 has protective effects on OM hepatocytes with up-regulation of PPARα[159]. TNFα reduces insulin receptor activation of IRS-1. This may be linked to disturbance of detergent resistant membrane micro domains in OM through an increase in the ganglioside GM3[160]. Leptin stimulates the release of TNFα and several other cytokines that affect metabolic functions[161]. However, OM have increased levels of TNFα[162] and OM made TNF deficient are less insulin resistant[163]. It is therefore unclear whether leptin deficiency affects the way cytokines can play a role in insulin resistance and other aspects of metabolism.

OM have adrenal hypertrophy and increased secretion of corticosteroids with normal diurnal rhythm[164,165,166]. One consequence of increased glucocorticoids is a diminished muscle glucose uptake[167]. Adrenalectomy reduces blood glucose and weight gain in both young[168] and adult OM[169], and leptin treatment reduced plasma corticosterone levels, probably because of inhibition of corticotropin-releasing factor neurons[167]. OM also have increased serum cholesterol[164,171]. Early studies reported no hypertriglyceridemia[19], which is consistent with a lipoprotein profile with elevated LDL and HDL, but low hepatic VLDL secretion[172,173,174]. However, most later studies suggest that there is also a modest hypertriglyceridemia[61,174,175].

Both insulin resistance and altered ß-cell function are secondary events to leptin deficiency in OM. The relatively mild "diabetes", despite severe insulin resistance, could speak in favor of a primary ß-cell defect being decisive for the development of type-2 diabetes. On the other hand, insulin resistance is the primary defect in the desert gerbil *Psammomys obesus* model for type-2 diabetes[176]. *P. obesus* and OM share many features in the initial stages including obesity, hyperinsulinemia, and hyperglycemia, but *P. obesus* are also hyperleptinemic and leptin resistant[173]. Both OM and *P. obesus* have an increased ß-cell sensitivity to glucose[177]. ß-Cell number and insulin release capacity is reduced with age in diabetes prone *P. obesus,* whereas islet size increases up to 6 months of age and insulin release is maintained in OM[73]. Islets from old OM show few signs of degeneration[28] and ß-cell replication can resume in old OM if they are again subjected to high glucose[63]. We do not know why the syndrome in OM does not progress to uncompensated diabetes and ß-cell destruction. The "diabetic" components observed in leptin-deficient humans appear to be rather mild[178,179] and the comparison with the desert gerbil indicates that the metabolic consequences of leptin deficiency can be more easily compensated for even with unlimited food intake. It is also evident from other experience in mice that not only leptin deficiency, but also genetic background plays a role because KsJ OM have impaired islet function[4] and increased lipotoxic islet damage[82] when compared with OM on a 6J background.

The OM metabolic condition causes redistribution of fat storage from fat to liver and other nonadipose tissues. This increased uptake and storage of lipids is thought to induce cell damage and apoptosis. OM hepatocytes have been used as a model system to explore mechanisms underlying initial phases of cytolipotoxicity. OM seldom gets severe liver damage. This can be because they have reduced inflammatory responses and reduced connective tissue formation when compared with normal mice. The condition in OM may be linked may be linked to low adiponektin and high TNF$\alpha$ levels or other strain-related distinctions in lipid handling. See Pertusa et al.[177] for review.

## IMMUNE SYSTEM

Overnutrition and obesity is coupled to changes in both the innate and the adaptive immune system, and the concept of "obesitis" has been put forward to describe the many similarities between obesity and inflammation[181]. Malnutrition also clearly affects the immune system in a negative way[182]. There are leptin receptors in all cell types of innate and adaptive immunity[183,184] and leptin affects most aspects of the immune system[185]. OM show differences from lean mice with regard to both defense against infection, rejection reactions, and inflammatory reactions[186,187]. Leptin restores the immune function of OM in parallel with metabolic functions. However, also less dramatic improvements of metabolic function, such as after arginine supplementation[188], stimulate immune function.

Leptin is a survival factor for T-cells during maturation in the thymus[189]. Both the number of T-lymphocytes[190] and the thymus and spleen weights[191] are reduced in OM. Leptin stimulates thymic growth[192] and OM do not have the same increase in T-cells as found in obesity induced by a high fat diet[193]. Several T-cell antigens are expressed aberrantly in OM[194] and leptin directly stimulates $CD4^+$ T-cells from OM[195]. T-cells produce leptin, but adipocyte-derived leptin is probably more important for immune regulation[196].

Phagocytotic activity is decreased in OM macrophages and is stimulated by leptin treatment[143,196,197]. Monocyte chemoattractant protein-1 is induced by insulin in adipose tissue and is increased in OM[198]. This may induce fat cell dedifferentiation and the phagocytic function of preadipocytes is reduced in OM[199]. OM also have impaired antigen-presenting cell function[200].

Beneficial effects of the reduced immune response in OM include lower concentrations of mannan-binding lectin, which can increase the risk of inflammatory disease[201], less severe arthritis after immunization[202], and less colitis after induction of intestinal inflammatory response[203]. OM are also partially protected against *Clostridium* toxin[55] and against concanavalin-A–induced hepatoxicity[204]. They have a delayed pulmonary response to bacterial infection, but are equally susceptible to disease[205,206]. Cardiac damage induced by viral myocarditis is larger in OM than in lean controls, probably due to a defective T-cell response[207].

## THE CARDIOVASCULAR SYSTEM

Leptin increases blood pressure, heart rate, and renal excretory function, most likely through an increased sympathetic nerve activity[208,209,210,211,212,213]. Those observations have mostly been done in rats and rabbits, but OM attest to this because OM have a reduced sympathetic nervous system activity[214]. When compared with normal lean mice, both lower[215] and higher[216] arterial pressure has been observed. The reduced SNS activity may also be an important factor for metabolic derangements[76], and OM may not be a model with hypertension coupled to obesity as part of a metabolic syndrome. The long-standing hyperglycemia of OM reduces vascular muscle contraction in response to glucose[217], but caloric restriction lowers blood pressure in OM as in normal mice, showing that this effect is only partly dependent on leptin[216].

The number of muscular arterioles is reduced in leptin-deficient mice[218]. Leptin receptors are present in endothelial cells and leptin induces angiogenesis in OM[219,220], possibly through the up-regulation of endothelial VEGF[221]. Ischemia-induced retinal neovascularization is markedly suppressed in 17-day-old leptin-deficient OM[221] and leptin stimulates retinal neovascularization. OM have impaired endothelial-dependent vasodilatation and this is also reversed by leptin replacement[222].

Leptin stimulates hematopoiesis through leptin receptors on hematopoietic stem cells[223,224,225,226,227]. There is a weak correlation between blood cell number and serum leptin levels in humans[228]. OM have reduced numbers of mononuclear cells[229], but they are polycythemic[230]. The increased number and size of red blood cells may be caused by the hyperinsulinemia[231]. A changed shape and size of red blood cells, and a reduced deformability is observed in OM, coinciding with a period of very high blood glucose[232,233].

Platelet thrombi form slowly and are unstable in *db/db*[234] and OM, and leptin potentiates the aggregation of OM platelets[235]. The fact that platelets have functional leptin receptors raises the possibility that the hyperleptinemia associated with obesity enhances the risk of atherothrombotic complications[236]. Inhibition of leptin reduced the risk of thrombosis in normal mice[237]. OM are protected from experimentally induced atherogenic insult to the vessel wall and this protection is lost after leptin treatment[238]. Plasminogen activator inhibitor-1 (PAI-1) is increased in serum from OM[239]. OM have perivascular fibrosis, which can be coupled to increased PAI-1 and TGFß[240].

Cardiomyopathy with abnormal diastolic left ventricular function is a finding in many diabetic patients[241]. OM has proven to be a good model for the study of this condition. Reduced myocardial efficiency is an early abnormality in OM[242,243] and disturbed OM cardiac myocyte metabolism precedes cardiomyopathy[242]. OM hearts have increased expression of enzymes that stimulate myocyte fatty acid uptake and triglyceride storage. They also have increased oxidative stress related to enzymes involved in contraction[244]. This is paralleled by cardiac diastolic dysfunction[245] and left ventricular hypertrophy, which is completely reversed by leptin treatment[246] or CNTF[247], but not by weight loss alone. There is also considerably increased cardiac apoptosis in OM[248]. However, in comparison with *db/db* mice, the OM have relatively small changes in cardiac muscle lysosomal enzymes involved in cardiomyopathy[249].

## OSTEOGENESIS AND CONNECTIVE TISSUE REPAIR

Obesity and increased body mass have been recognized to “protect” from bone loss and the risk of fracture[250,251]. Leptin levels correlate with fat mass and leptin stimulates bone formation, but there are conflicting data regarding the correlation between serum leptin levels and bone mass and bone formation in humans[252,253,254,255], particularly when the effect of leptin is analyzed independently of fat mass. It has been debated whether the protective effect of increased body weight is related to fat or muscle mass[250,251]. Leptin-deficient OM have lower bone mass in long bones and reduced bone formation when compared with lean mice[256,257], and this could be restored with leptin treatment[256]. The changes in long bones were observed, particularly at young ages[258,259], and could be related to the low muscle mass[254]. Growth plates of OM have a reduced collagen expression and disturbed collagen fibril arrangement, causing the growth plate to be more vulnerable[260].

Leptin-induced signaling was observed in both osteoblasts and chondrocytes, indicating direct peripheral effects of leptin[253,254,261], but several studies in OM point to a central effect of leptin in stimulating bone formation[262,263,264]. However, vertebrate bone mass is increased in OM[259,262]. This may reflect the balance between stimulatory effects of leptin on both bone formation and bone resorption. The expression of cocaine amphetamine regulated transcript (CART) in the CNS is controlled by leptin and nearly abolished in OM. CART signaling may inhibit RANK ligand expression in osteoblasts and thereby inhibit osteoclast differentiation[265]. OM may have increased bone resorption because leptin inhibition of CART signaling is not present[265]. OM also develop abnormal incisor teeth coinciding with reduced food intake and lowering of body weight at 6–8 months[266].

Skin from OM has a low tensile strength and there is impaired wound healing probably because of reduced collagen deposition[267,268**].** Leptin restores the wound healing by increasing fibroblast function, but probably only to a small degree by the increased angiogenesis[269,270]. Systemically and topically supplemented leptin improves re-epithelialization in OM[271]. The effect of leptin on keratinocytes is through the STAT-3 pathway[271,272]. There is less hepatic fibrosis in OM after toxic injury or *Schistosoma mansoni* infection, but fibrosis is observed when the animals have been treated with leptin[273]. The hepatic stellate cells that also account for most of the collagen production produce leptin, and TGFß may mediate the effect of leptin[273,274]. Nitric oxide synthesis is important for normal skin repair. Leptin deficiency is accompanied by dysregulated nitric oxide synthesis, which can be restored by leptin treatment[275].

## MALIGNANCIES

Leptin stimulates growth and metastasis in several types of malignancies[276,277,278]. It was early recognized that OM have a lower incidence of many types of malignancies including pulmonary and breast cancer[279,280]. One exception was hepatomas that were more prevalent in OM[280]. Some tumor types like melanoma grow faster in OM, but here there is also a resistance to metastasis formation when compared with lean mice[281,282]. The resistance to metastasis is not fully explained, but probably involves reduced angiogenesis as well as reduced tissue turnover because of increased PAI-1.

## REPRODUCTION

The physiological role of leptin in reproduction may be to signal an adequate energy supply for reproductive function[283,284]. The leptin deficiency results in a form of hypogonadotropic hypogonadism[285]**,** and both male and female OM are infertile. Leptin restitution restores both puberty and fertility in OM[285,286,287]**.** Male OM have impaired spermatogenesis and increased germ cell apoptosis[288], but partial restoration of fertility in male OM is sometimes observed when the mice are cross-bred into another genetic background[289] or after food restriction[290]. A number of early[291] and later studies[292] show that fertility can also be restored by the substitution with gonadotropic hormones.

Leptin regulates food intake and body weight through inhibition of hypothalamic NPY orexigenic signals and stimulation of MSH (MC4) and other anorexigenic signals. Central effects of leptin on reproduction probably involve inhibition of NPY (Y4), but the role of stimulatory neuropeptides is under debate[12,293,294,295]. Recent studies in OM strongly suggest that kisspeptin/GPR54 signaling is important for the central effects of leptin[296,297]. Leptin plays a role in the initiation of puberty in mice, but this effect may be permissive rather than actually triggering the onset of puberty[298].

The OM metabolic condition as such can affect reproductive organs because cytolipotoxicity of endometrial and ovarian follicular cells is seen in OM older than 4 weeks[299,300,301]. However, leptin may have direct peripheral effects since there are leptin receptors in ovary and decidua, in mature oocytes, and in trophoblast cells[302,303]. The peripheral effects of leptin may include sensitizing the follicles to chorionic gonadotropin[304], but leptin can also induce ovulation by activation of ovarian metalloproteinases independently of hypothalamic GnRH[305]. Leptin is not required for pregnancy and parturition in OM once implantation is established[306].
